# MR-based radiomics of mesorectal fat for improved prediction of perirectal lymph node metastasis and extramural venous invasion in locally advanced rectal cancer

**DOI:** 10.1371/journal.pone.0357268

**Published:** 2026-09-02

**Authors:** Yaniga Swaengdee, Sararas Khongwirotphan, Jaravee Lasode, Phakakarn Kuecharoen, Phathayphout Phetvilay, Thitithep Limvorapitak, Anapat Sanpavat, Sira Sriswasdi, Piyaporn Boonsirikamchai, Yothin Rakvongthai

**Affiliations:** 1 Medical Physics Program, Department of Radiology, Faculty of Medicine, Chulalongkorn University, Bangkok, Thailand; 2 Chulalongkorn Biomedical Imaging Group, Department of Radiology, Faculty of Medicine, Chulalongkorn University, Bangkok, Thailand; 3 Department of Radiological Technology and Medical Physics, Faculty of Allied Health Sciences, Chulalongkorn University, Bangkok, Thailand; 4 Division of Diagnostic Radiology, Department of Radiology, King Chulalongkorn Memorial Hospital, Bangkok, Thailand; 5 Division of Diagnostic Radiology, Department of Radiology, Faculty of Medicine, Chulalongkorn University, Bangkok, Thailand; 6 Colorectal Division, Department of Surgery, Faculty of Medicine, Chulalongkorn University, Bangkok, Thailand; 7 Department of Pathology, Faculty of Medicine, Chulalongkorn University, Bangkok, Thailand; 8 Center for Artificial Intelligence in Medicine, Research Affairs, Faculty of Medicine, Chulalongkorn University, Bangkok, Thailand; 9 Center of Excellence in Computational Molecular Biology, Chulalongkorn University, Bangkok, Thailand; 10 Division of Nuclear Medicine, Department of Radiology, Faculty of Medicine, Chulalongkorn University, Bangkok, Thailand; Fujita Health University: Fujita Ika Daigaku, JAPAN

## Abstract

**Objective:**

Accurately assessing residual disease after neoadjuvant chemoradiotherapy (nCRT) in locally advanced rectal cancer (LARC) remains challenging. Residual extramural venous invasion (EMVI) and perirectal lymph node (PLN) metastasis indicate adverse outcomes, but treatment-related changes obscure their detection on post-treatment MRI. This study developed MRI-based radiomics models to predict residual EMVI and PLN metastasis using post-nCRT restaging MRI.

**Materials and methods:**

In this retrospective study, 219 patients with LARC who completed nCRT and underwent post-treatment MRI for restaging prior to surgery were included. Radiomic features were extracted from manually segmented regions of interest encompassing the primary tumor and mesorectal fat on high-resolution T2-weighted images using PyRadiomics. Logistic regression (LR), support vector machine (SVM), and random forest (RF) models were developed to predict pathological EMVI and PLN status. Model performance was assessed using repeated 5-fold cross-validation, with the area under the receiver operating characteristic curve (AUC) as the primary evaluation metric. Differences in model performance were compared using DeLong test.

**Results:**

For EMVI prediction, the combined tumor and mesorectal fat radiomics model achieved the highest AUC of 0.797 ± 0.073 using the LR model. For PLN prediction, the combined model also demonstrated superior performance, achieving an AUC of 0.824 ± 0.073. Models incorporating both tumor and mesorectal fat features consistently outperformed single-region models.

**Conclusion:**

MRI-based radiomics models using post-nCRT restaging images could predict residual EMVI and PLN metastasis in LARC. Incorporating mesorectal fat features improved model performance, suggesting that information from the surrounding mesorectal compartment may be useful for post-treatment risk assessment.

## Introduction

Colorectal cancer (CRC) is one of the major malignancies of concern, according to the World Health Organization (WHO). Globally, CRC ranks as the third most common cancer and the second leading cause of cancer-related mortality [1]. In 2020, more than 1.9 million new cases were diagnosed, along with over 900,000 deaths. Fortunately, the incidence rate of CRC has declined due to improvements in diagnostic technology. In contrast, the incidence rates of CRC are rising in Asia, especially in East and Southeast Asia, largely due to the consumption of spicy foods and smoking habits. [2]

Locally advanced rectal cancer (LARC) is the extension of tumor beyond the rectal wall [3]. Extramural venous invasion (EMVI) and perirectal lymph node (PLN) status are important pathological factors in determining prognosis. Both EMVI and PLN are strongly related with increased recurrence rates and mortality in LARC patients, emphasizing their essential role in patient management and outcome prediction [4–7].

Extramural venous invasion (EMVI), defined as the spread of tumor cells into veins outside the muscularis propria, significantly impacts recurrence-free survival, systemic metastasis, cancer-specific mortality, and regional lymph node metastasis in LARC patients [4,5,8]. Its presence is recognized as an indicator of poor long-term outcomes. Total mesorectal excision is the gold standard for confirming both EMVI and PLN status, a surgery involving the precise excision of the mesorectal fascia and fat [3].

However, the treatment paradigm for LARC has evolved substantially in recent years. With the increasing use of neoadjuvant chemoradiotherapy (nCRT) and total neoadjuvant therapy, there has been a shift from routine radical surgery toward organ-preserving strategies in selected patients. In particular, the “watch-and-wait” approach has emerged as a viable alternative for patients who achieve a clinical complete response after neoadjuvant treatment. This strategy aims to preserve anorectal function and avoid surgical morbidity without compromising oncologic outcomes, and has been supported by prospective studies and contemporary clinical guidelines [9,10].

As organ preservation becomes more widely adopted, accurate post-treatment assessment is increasingly critical. Restaging MRI plays a central role not only in evaluating primary tumor response but also in identifying residual disease within the mesorectum. Recent expert consensus highlights that assessment of both tumor response and nodal status is essential when determining eligibility for a watch-and-wait strategy, particularly in distinguishing complete or near-complete responders from those with residual disease [11].

Among post-treatment risk factors, residual viable EMVI and persistent perirectal nodal disease are of particular clinical importance. Persistent EMVI following neoadjuvant therapy has been shown to correlate with increased risk of distant metastasis and poorer survival outcomes [12,13]. Similarly, residual nodal metastases may remain despite significant tumor regression and are associated with higher recurrence rates and worse prognosis [14,15]. These features are especially critical in the context of a watch-and-wait approach, as failure to detect residual mesorectal disease may result in inappropriate selection of patients for non-operative management.

Magnetic resonance imaging (MRI) has emerged as a crucial tool in tumor staging [16,17] and the non-invasive evaluation of EMVI and PLN status, offering superior spatial resolution compared to computed tomography (CT) [18]. High-resolution T2-weighted MRI is particularly valuable in visualizing tumor invasion due to its excellent contrast resolution. However, the accurate identification of EMVI on MRI remains highly dependent on radiologist expertise [8,19], and while MRI can detect PLN status, it cannot definitively confirm nodal metastasis [20].

To overcome limitations associated with subjective image interpretation, quantitative approaches such as radiomics are gaining prominence in medical imaging. Radiomics involves the extraction of high-dimensional quantitative features from medical images [21], allowing for reproducible, observer-independent analysis. This methodology has shown significant potential in predicting clinical outcomes across various cancers.

Radiomics research in locally advanced rectal cancer (LARC) has advanced significantly; however, most studies have focused predominantly on tumor characteristics, often overlooking the critical role of peritumoral tissues, such as mesorectal fat. [16,22–25]. Mesorectal fat, analyzed during mesorectal excision, has been linked to poor outcomes in LARC patients and holds significant prognostic value [26–28]. Integrating radiomic features from both the tumor and surrounding mesorectal fat may provide a more comprehensive predictive model for EMVI and PLN status. While prior radiomics studies have examined mesorectal fat, these have been limited to evaluating pathologic response rather than exploring its prognostic potential for EMVI and PLN prediction [29], underscoring the novelty and importance of the present study.

This study aims to develop and validate radiomics-based models leveraging MRI data from LARC patients who completed neoadjuvant chemoradiotherapy and underwent post-treatment MRI to predict residual EMVI and PLN status. By incorporating quantitative features from both the tumor and mesorectal fat, the study seeks to enhance prognostic accuracy and advance personalized treatment strategies for LARC.

## Materials and methods

### Data collection

This study received approval from the Institutional Review Board of the Faculty of Medicine, Chulalongkorn University, Bangkok, Thailand. (IRB No. 0555/67) The requirement for informed consent was officially waived by the IRB due to the retrospective nature of the study. All patient data, including pathological statuses and MRI images, were entirely de-identified and anonymized prior to analysis to ensure that individual patient identities cannot be traced back.

A retrospective cohort comprising 219 patients diagnosed with locally advanced rectal cancer (LARC) was collected from King Chulalongkorn Memorial Hospital between January 1, 2013, and April 5, 2023. The eligibility criteria for inclusion were defined as follows:

The patients with LARC who had completed nCRT and underwent post treatment MRI were included. Only those with high-resolution T2-weighted MRI images of sufficient quality for analysis were considered.

Exclusion criteria included patients whose interval between post-nCRT MRI and surgical treatment exceeded 90 days. Additionally, MRI images of poor quality that were unsuitable for segmentation or analysis were excluded. Patients with pathological diagnoses that did not meet standard criteria and those with pathologically confirmed mucinous adenocarcinoma were also not included in the study.

MRI images were acquired using different scanner models. For 1.5T MRI scanners, the Siemens Magnetom Aera (Siemens Medical Solutions, Forchheim, Germany) and the GE SIGNA Artist (GE Medical Systems, Milwaukee, USA) were used. For 3.0T MRI scanners, the Philips Ingenia Elition (Philips Healthcare, Best, Netherlands) and the GE SIGNA HDxt (GE Medical Systems, Milwaukee, USA) were utilized. Patient data were retrospectively retrieved from institutional records. Imaging data were extracted from the Picture Archiving and Communication System (PACS), and pathological data, including PLN and EMVI status, were obtained from the Hospital Information System (HIS). Only high-quality high-spatial-resolution T2-weighted MRI images in the axial oblique plane, oriented perpendicular to the long axis of the rectal tumor or the area impacted by treatment were included for analysis.

After data collection, a total of 219 cases were included in the study. For EMVI status, 72 cases were pathologically confirmed as positive and 147 cases as negative. For PLN status, 63 cases were confirmed as positive and 156 cases as negative.

### Image segmentation

**Fig 1:**
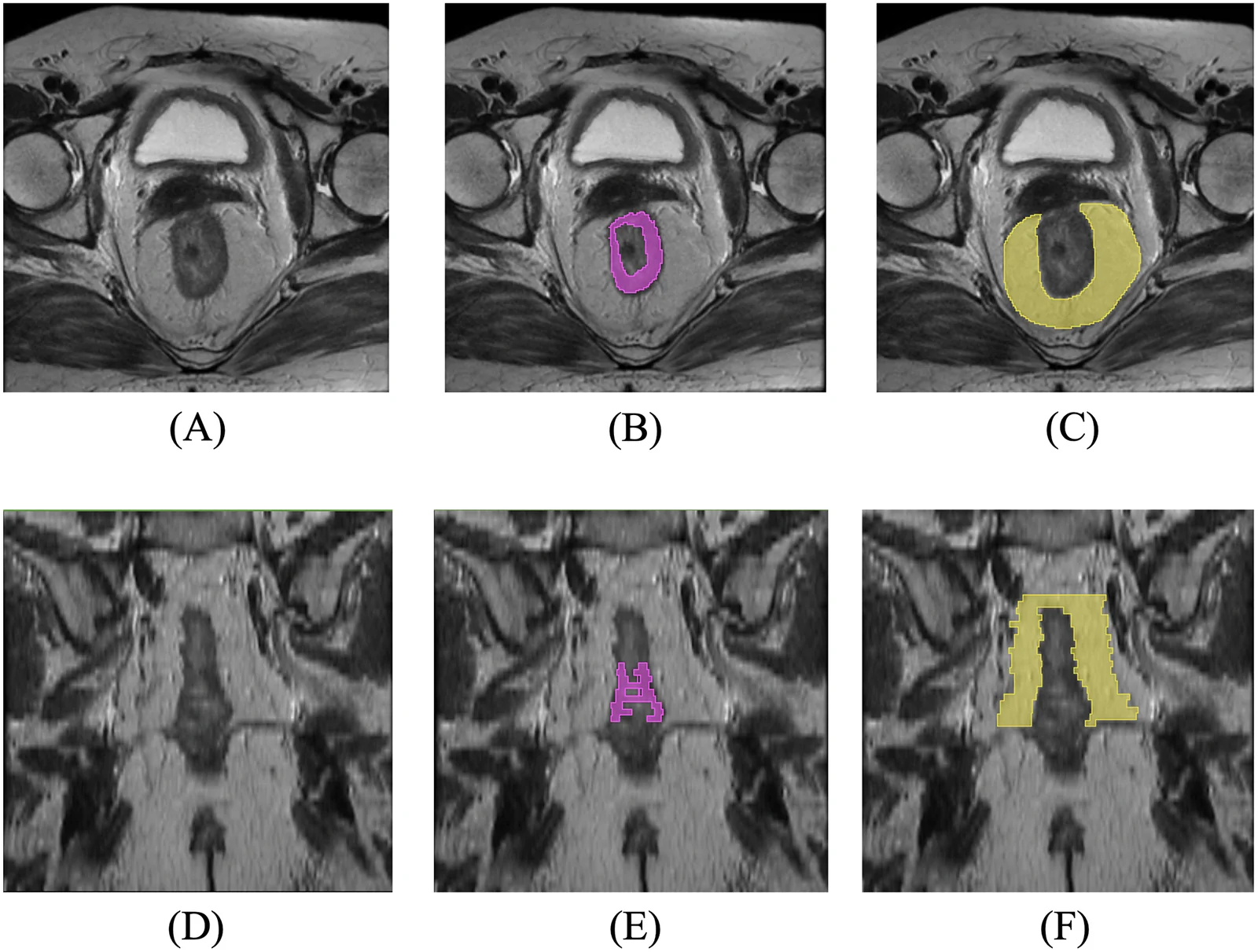
Example of tumor and mesorectal fat ROI segmentation on post nCRT T2-weighed MRI. (A) and (D): The axial oblique and coronal oblique of T2-weighted MRI demonstrating circumferential primary rectal tumor. (B) and (E): Corresponding slices showing segmentation of rectal tumor (pink contour). (C) and (F): Corresponding slice showing segmental of mesorectal fat ROI (yellow contour) with the cranio-caudal extent of the merorectal ROI covering the mesorectal compartment approximately 5 cm proximal to the tumor level and the whole mesorectal rectum at below the tumor level.

The region of interest (ROI) for this study encompassed the mesorectal fat, covering at least 5 cm superior and 5 cm inferior to the tumor margin, if feasible, to ensure adequate coverage of the mesorectal compartment at risk. This approach is supported by prior work from Brown et al. [30] and the MERCURY study group [31]. The ROI of the primary tumor was also separately obtained. Manual segmentation of the ROI on T2-weighted axial oblique MRI images was conducted using 3D Slicer version 5.0.3 on Windows by a board-certified radiologist. These ROIs are displayed in Fig 1.

To assess the robustness and inter-observer reproducibility of radiomic features, the 219 patients were distributed among three radiologists with 13, 7, and 4 years of experience, respectively. Each patient was manually delineated by one assigned radiologist to generate the primary tumor ROI used for radiomics feature extraction. To evaluate inter-observer reproducibility, a random subset of 30 patients was selected, and the two radiologists who had not performed the original delineation independently segmented the same tumors. Consequently, each of these 30 patients had three independent segmentations, which were used to calculate the intraclass correlation coefficient (ICC) to assess segmentation consistency across observers. Only the primary ROI for each patient was used for subsequent radiomics feature extraction and machine learning analysis, whereas the additional segmentations were used exclusively for ICC analysis.

### Radiomics feature extraction

Following segmentation, MRI images were resampled to an isotropic voxel size of 0.5 × 0.5 × 0.5 mm³ to minimize variability in radiomic feature extraction associated with heterogeneous image resolution across scanners [32]. The resampling resolution was selected based on the smallest voxel dimension (0.5 mm) in the original dataset, with remaining dimensions adjusted accordingly to generate isotropic voxels, a commonly recommended preprocessing step to improve feature reproducibility. Radiomic features were extracted from the resampled images using PyRadiomics version 3.1.0, implemented in Jupyter Notebook version 7.0.7 with Python version 3.12.1. A bin width of 0.3 was applied, yielding a bin count range of [32, 75]. A total of 1,288 radiomic features, including first-order features, shape and size features, texture-based features and filtered-based features were extracted for further analysis.

### Feature selection

**Fig 2:**
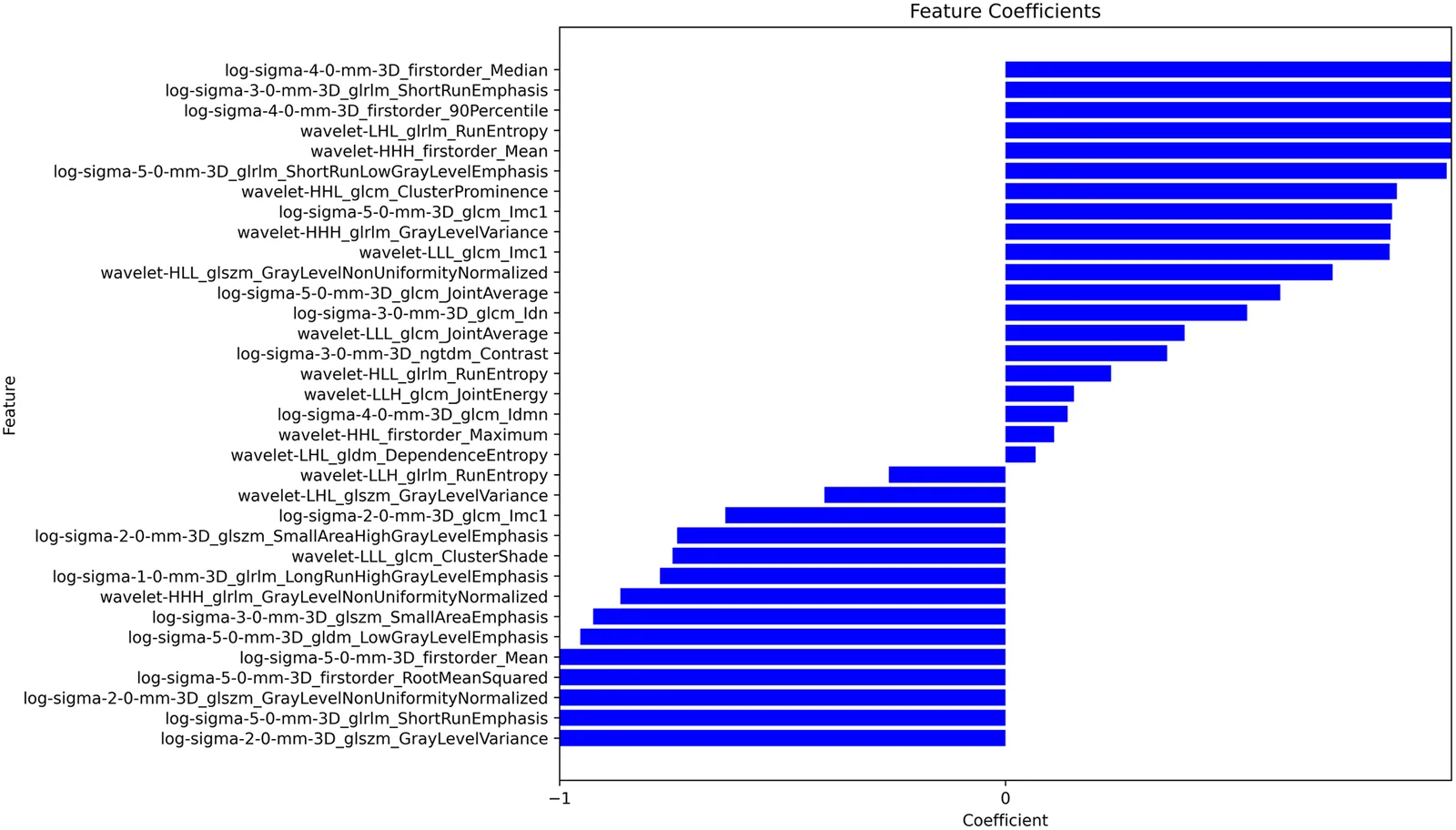
Features coefficient of the best performance model for EMVI prediction.

**Fig 3:**
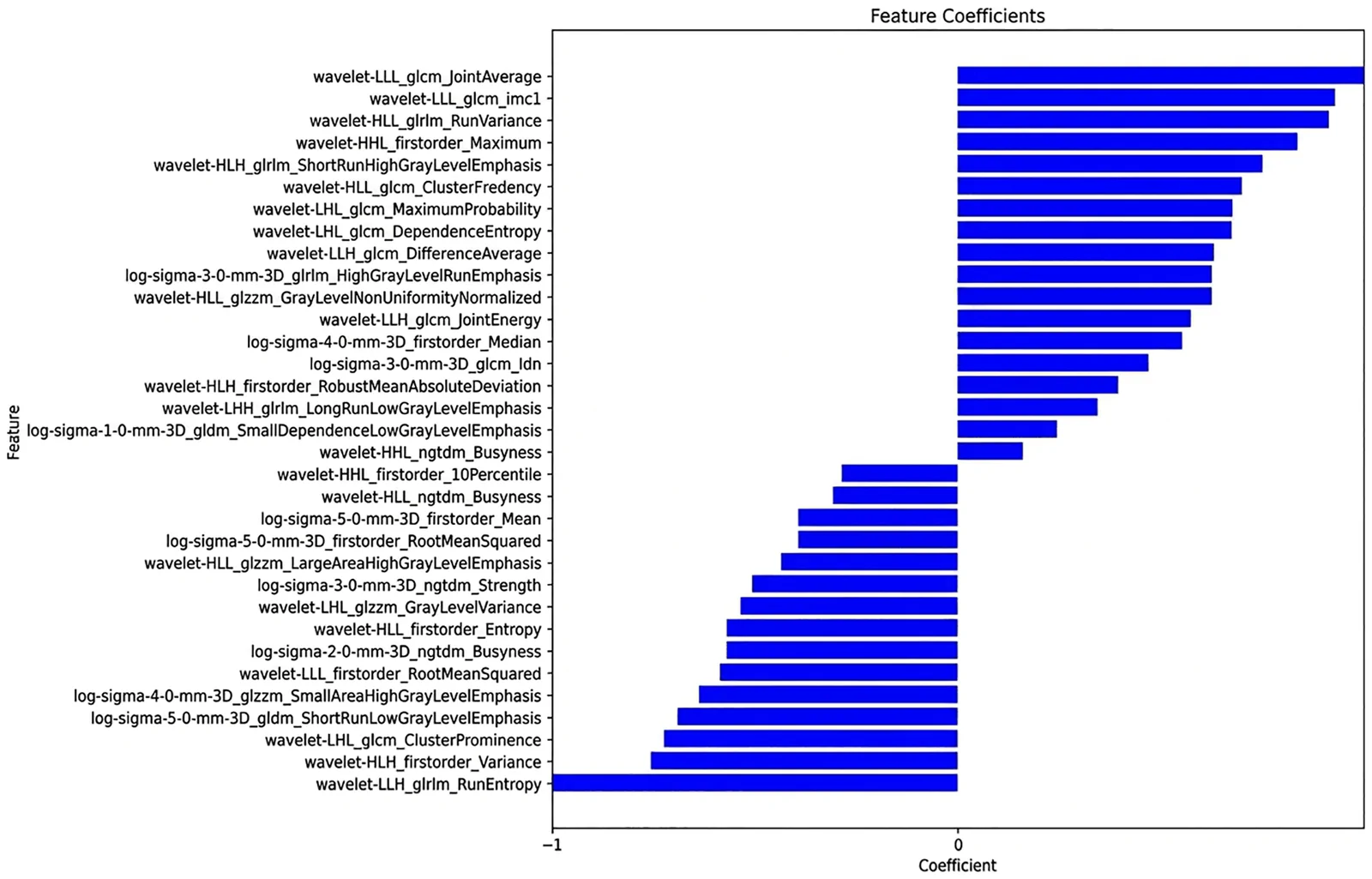
Features coefficient of the best performance model for PLN prediction.

Feature selection was performed in a structured, multi-step process to ensure reproducibility and clinical relevance:

1. Intraclass correlation coefficients (ICCs) were calculated from 30 independently segmented cases to evaluate feature reproducibility. Features with ICC values greater than 0.5 were deemed acceptable for further analysis.
2. Univariate logistic regression was employed to identify associations between radiomic features and pathological outcomes (PLN and EMVI status). The top 20% of features with the highest area under the receiver operating characteristic (AUC) scores and ICC > 0.5 were shortlisted.
3. Recursive Feature Elimination with 5-fold cross-validation (RFECV) was applied to refine the feature set. The model used for feature selection in each case matched the corresponding predictive model. To mitigate underfitting and overfitting, the final model included 15–20 features (as shown in Figs 2 and 3), selected based on optimal AUC performance.

### Model building and statistical analysis

Following ICC filtering, univariate analysis, and recursive feature elimination with cross-validation (RFECV), the final radiomics feature subset was identified using the complete cohort. Hyperparameter optimization was subsequently performed using a grid-based search strategy over 20 repetitions of 5-fold cross-validation (100 train-validation splits) and the resulting models and corresponding performance statistics were reported.

Three predictive models were developed and evaluated: Logistic Regression (LR), Random Forest Classifier (RF) and Support Vector Machine (SVM) with a radial basis function (RBF) kernel.

Hyperparameter tuning was performed for each model to optimize predictive performance. For the LR model, both the type of regularization penalty (L1 and L2) and the regularization strength parameter (C) were systematically tuned. In the SVM model utilizing a RBF kernel, the kernel coefficient (gamma) and the regularization parameter (C) were adjusted. For the RF model, key hyperparameters including the number of estimators, maximum tree depth, and minimum number of samples required at a leaf node were optimized.

Model performance was evaluated using classifier-predicted probabilities, with the area under the receiver operating characteristic curve (AUC) as the primary evaluation metric. Statistical comparisons between models were performed using a modified DeLong test. Briefly, pairwise DeLong tests were conducted between competing models for each round using classifier-predicted probabilities, from which ROC-AUC values were derived. Since performance evaluation was based on repeated resampling, the resulting statistics across 100 iterations were combined using an inverse-variance-weighted approach [33]. This approach was selected because a conventional single DeLong test is designed for ROC comparison on a single dataset and may not adequately account for variability introduced by repeated resampling procedures. To control for false discovery rates arising from multiple comparisons, the Benjamini–Hochberg procedure was applied. All statistical analyses were conducted using Python-based tools.

## Results

### Model performance

For EMVI prediction, the Logistic Regression (LR) model demonstrated the highest overall performance compared with the Support Vector Machine (SVM) and Random Forest (RF) models across all feature sets. Using mesorectal fat radiomic features, the LR model achieved an AUC of 0.776 ± 0.073 (95% CI: 0.762–0.791), whereas the SVM and RF models achieved AUCs of 0.711 ± 0.073 (95% CI: 0.696–0.725) and 0.685 ± 0.075 (95% CI: 0.670–0.700), respectively. For the tumor radiomics model, the LR model achieved an AUC of 0.757 ± 0.079 (95% CI: 0.741–0.773), compared with 0.732 ± 0.074 (95% CI: 0.718–0.747) for SVM and 0.680 ± 0.077 (95% CI: 0.665–0.696) for RF model. When mesorectal fat and tumor radiomic features were combined, the highest performance was again observed for the LR model, achieving an AUC of 0.797 ± 0.073 (95% CI: 0.783–0.812), followed by the SVM model with an AUC of 0.726 ± 0.078 (95% CI: 0.710–0.741) and the RF model with an AUC of 0.725 ± 0.072 (95% CI: 0.711–0.740), as shown in Table 1.

**Table 1:** Model Performance for EMVI Prediction Using Mesorectal Fat, Tumor, and Combined Radiomic Features.

	Mesorectal Fat		
	Train	Validation	Features
LR	0.851 ± 0.017	0.776 ± 0.073	17
SVM	0.822 ± 0.016	0.711 ± 0.073	16
RF	0.991 ± 0.004	0.685 ± 0.075	18
	Tumor		
LR	0.846 ± 0.015	0.757 ± 0.079	17
SVM	0.820 ± 0.018	0.732 ± 0.074	16
RF	0.906 ± 0.010	0.680 ± 0.077	19
	Combined		
LR	0.930 ± 0.012	0.797 ± 0.073	34
SVM	0.845 ± 0.017	0.726 ± 0.078	32
RF	0.994 ± 0.003	0.725 ± 0.072	37

For PLN prediction, a similar trend was observed, with the LR model consistently achieving the highest performance across feature sets. Using mesorectal fat radiomic features, the LR model achieved an AUC of 0.758 ± 0.080 (95% CI: 0.740–0.772), while the SVM and RF models achieved AUCs of 0.729 ± 0.071 (95% CI: 0.715–0.743) and 0.726 ± 0.080 (95% CI: 0.710–0.741), respectively. In the tumor radiomics model, the LR model achieved an AUC of 0.806 ± 0.065 (95% CI: 0.793–0.819), compared with 0.764 ± 0.063 (95% CI: 0.751–0.776) for SVM and 0.712 ± 0.082 (95% CI: 0.694–0.727) for RF model. In the combined model incorporating mesorectal fat and tumor radiomic features, the highest performance was again observed for the LR model, achieving an AUC of 0.824 ± 0.073 (95% CI: 0.809–0.838), followed by the SVM model with an AUC of 0.789 ± 0.065 (95% CI: 0.776–0.802) and the RF model with an AUC of 0.777 ± 0.071 (95% CI: 0.763–0.791), as shown in Table 2.

**Table 2:** Model Performance for PLN Prediction Using Mesorectal Fat, Tumor, and Combined Radiomic Features.

	Mesorectal Fat		
	Train	Validation	Features
LR	0.840 ± 0.017	0.758 ± 0.080	16
SVM	0.856 ± 0.018	0.729 ± 0.071	18
RF	1.000 ± 0.000	0.726 ± 0.080	16
	Tumor		
LR	0.880 ± 0.015	0.806 ± 0.065	17
SVM	0.838 ± 0.015	0.764 ± 0.063	18
RF	1.000 ± 0.000	0.712 ± 0.082	16
	Combined		
LR	0.941 ± 0.010	0.824 ± 0.073	33
SVM	0.946 ± 0.010	0.789 ± 0.065	36
RF	1.000 ± 0.000	0.777 ± 0.071	32

### Model comparison

For both EMVI and PLN predictions, the Logistic Regression (LR) model demonstrated the best overall performance compared to the other models across all feature sets: mesorectal fat, tumor, and combined radiomics models.

**Fig 4:**
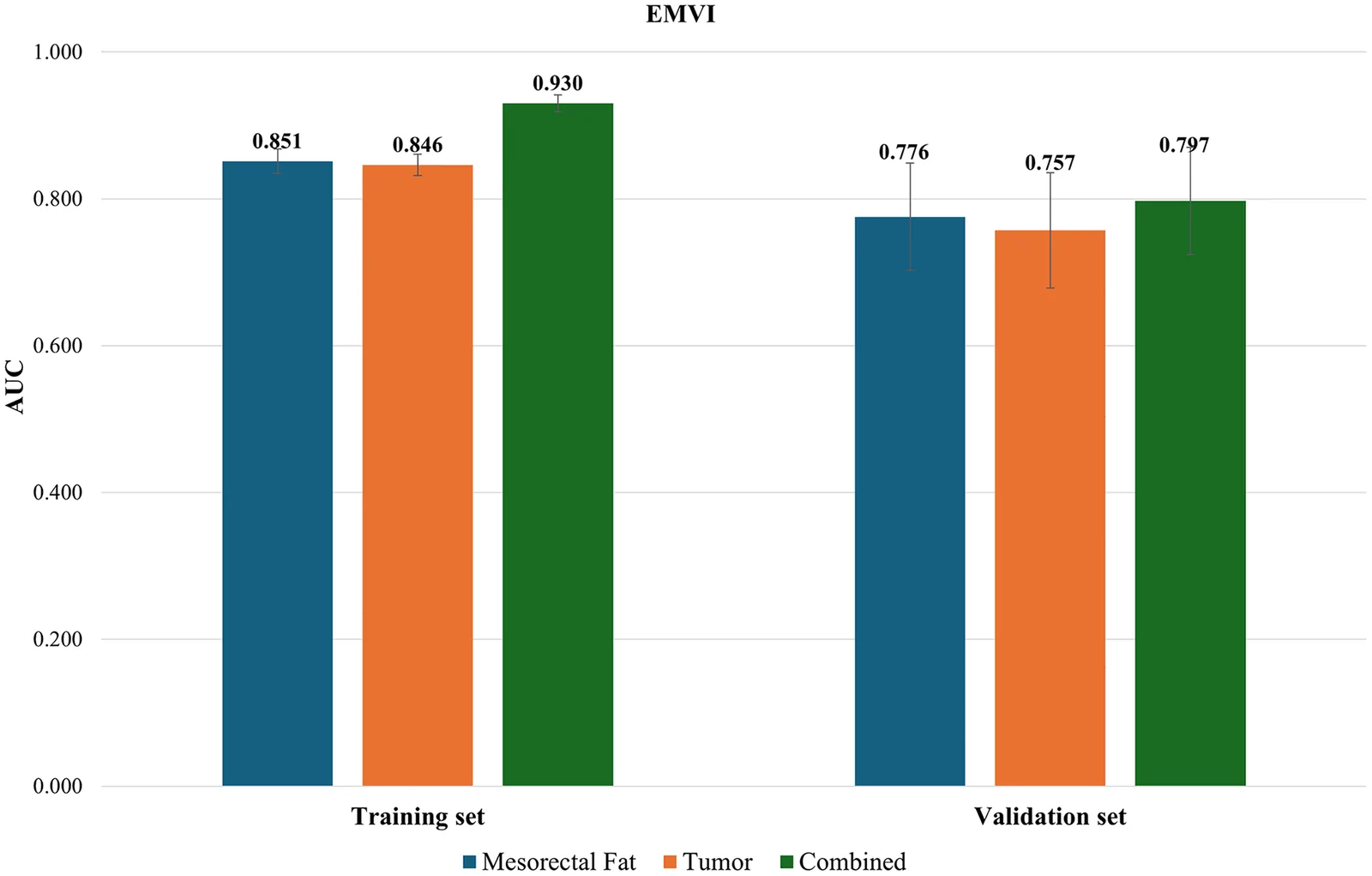
The Logistic Regression Model performance for EMVI Prediction in the Training and Validation Sets Across Mesorectal Fat, Tumor, and Combined Radiomic Features.

**Fig 5:**
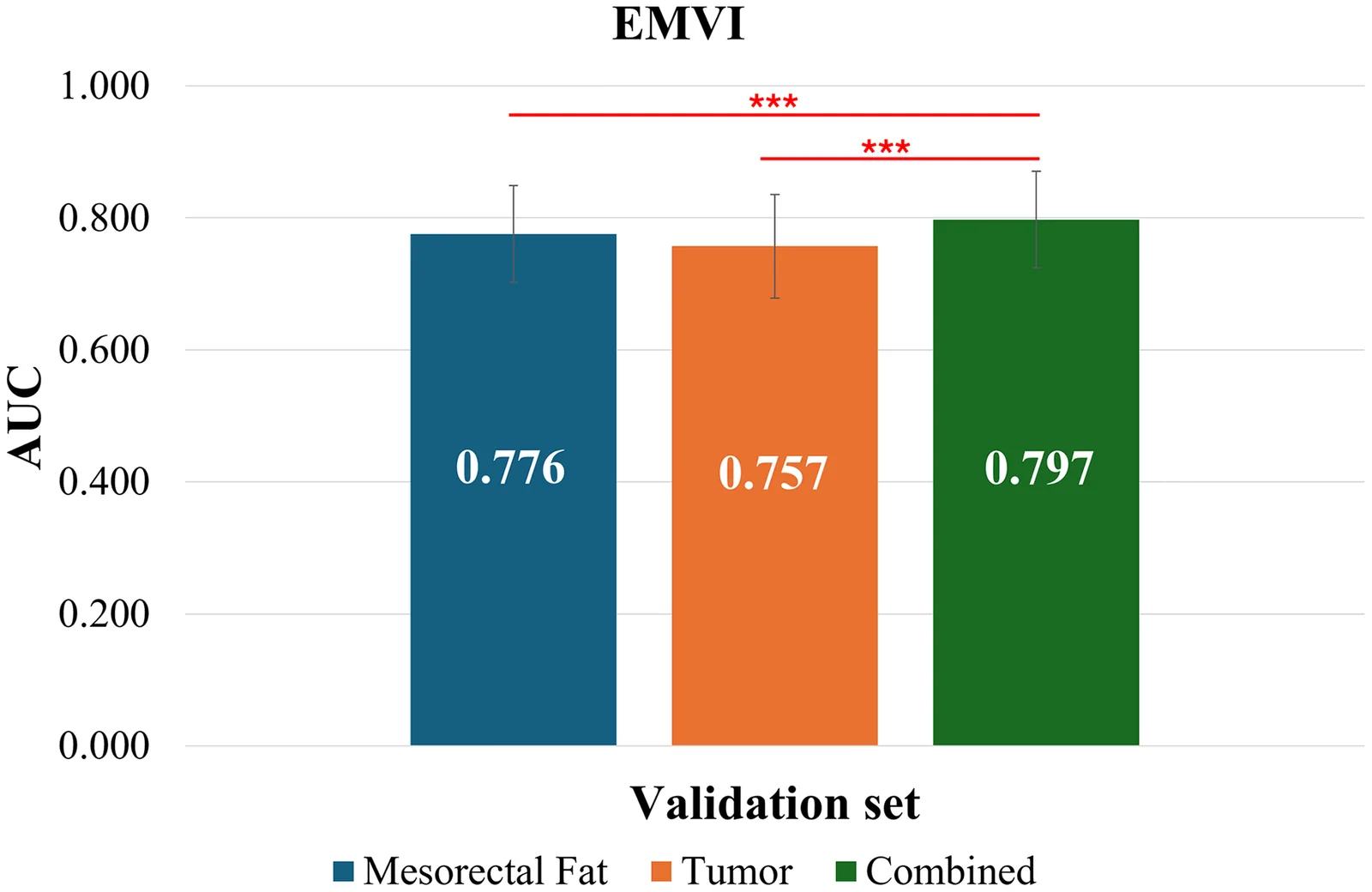
The comparison of best model performance between radiomics model from mesorectal fat, tumor and combined in EMVI prediction. *** indicates statistically significance difference (p-value < 0.05).

For EMVI prediction, the LR model achieved an AUC of 0.776 ± 0.073 from mesorectal fat radiomics features and 0.757 ± 0.079 from tumor radiomics features. When combining both features from mesorectal fat and tumor regions, the performance significantly improved, with the LR model achieving an AUC of 0.797 ± 0.073, as shown in Fig 4. Statistical analysis confirmed that the combined model was significantly superior to the individual models (p-value = 3.02 × 10^−13^ compared with the tumor model and p-value = 6.91 × 10^−12^ compared with the mesorectal fat model), as shown in Fig 5.

**Fig 6:**
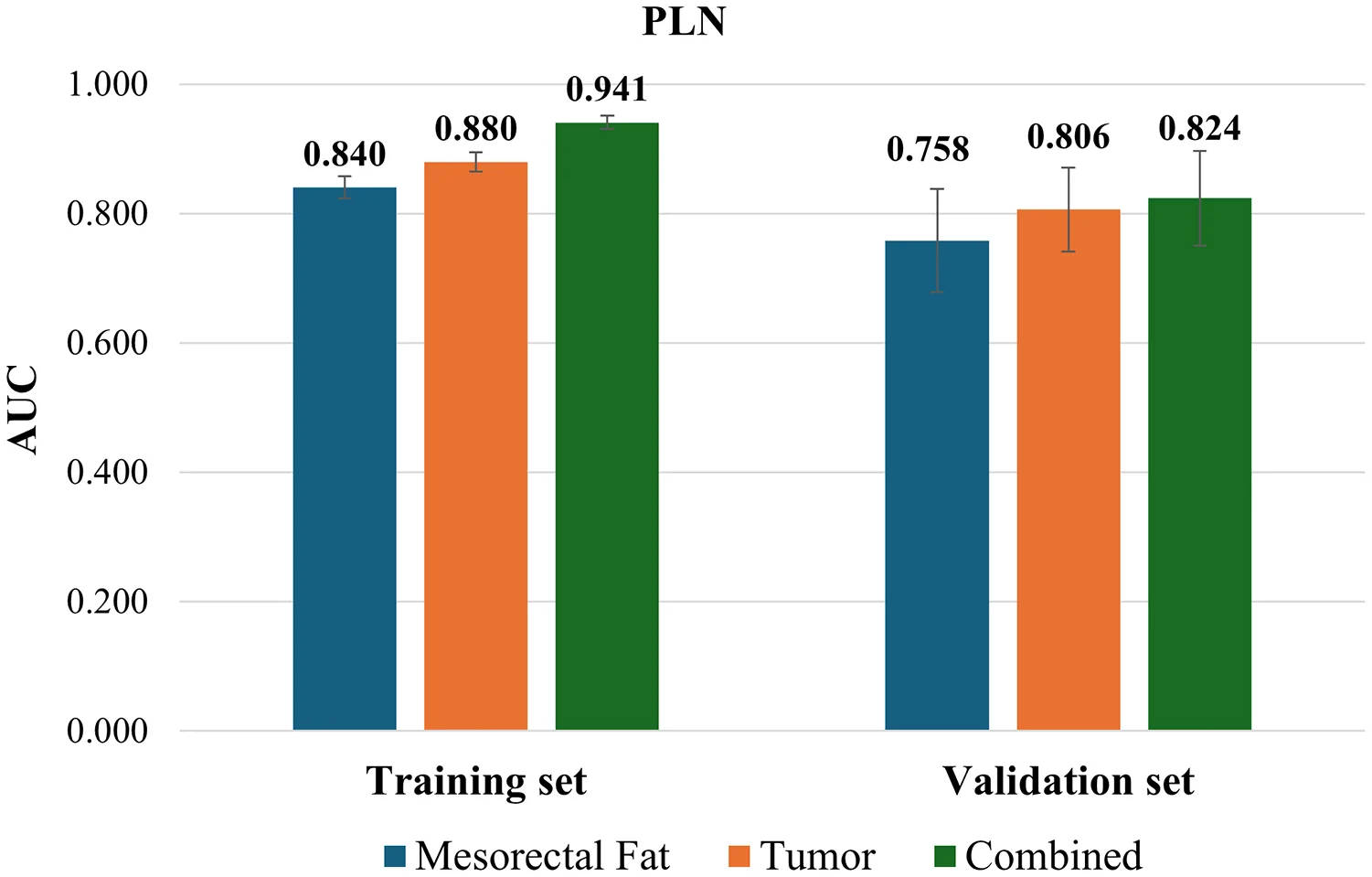
The Logistic Regression Model performance for PLN prediction in the training and validation sets across mesorectal fat, tumor, and combined radiomic features.

**Fig 7:**
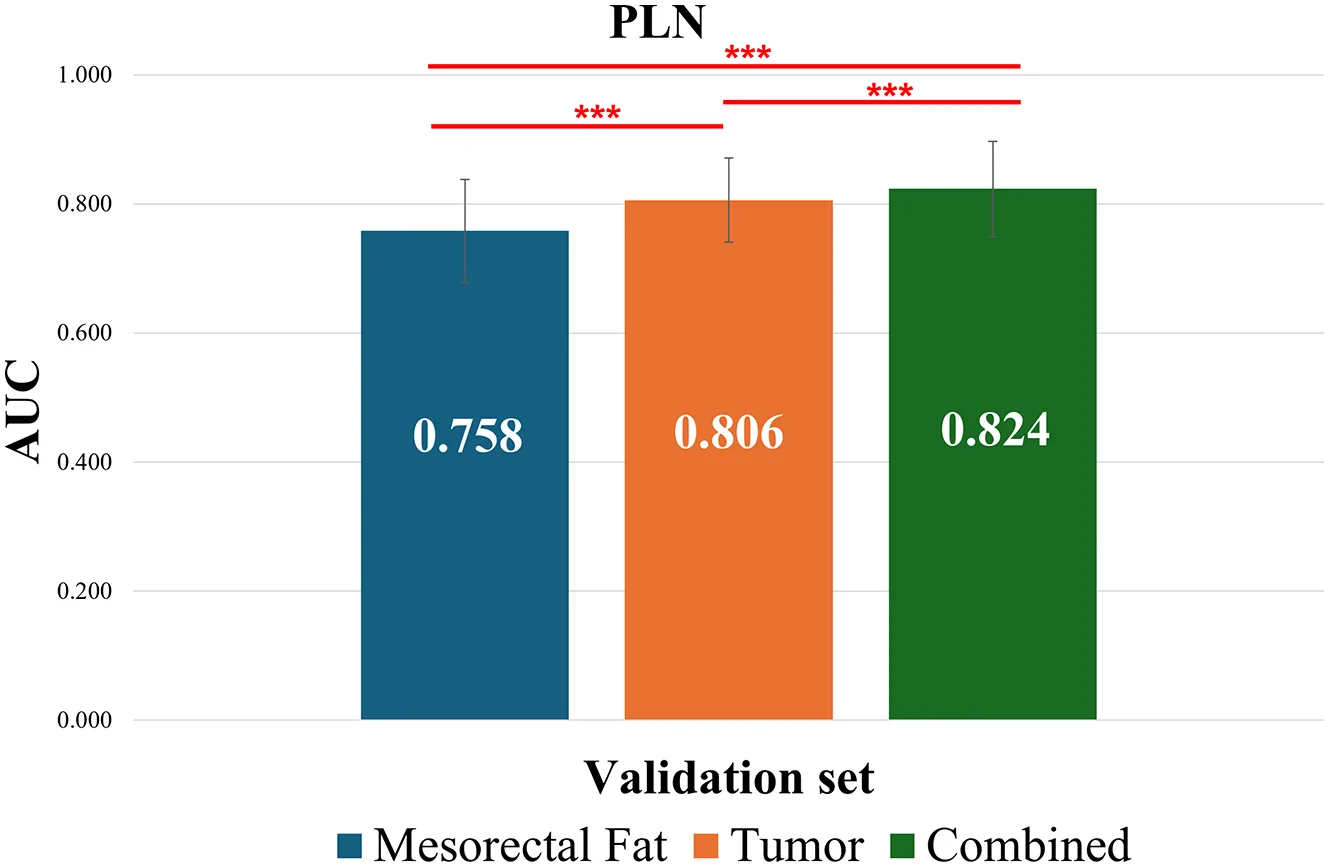
The comparison of best model performance between radiomics model from mesorectal fat, tumor and combined in PLN prediction. *** indicates statistically significance difference (p-value < 0.05).

For PLN prediction, the LR model achieved an AUC of 0.758 ± 0.080 (95% CI: 0.740–0.772) with mesorectal fat radiomics features and 0.806 ± 0.065 (95% CI: 0.793–0.819) with tumor radiomics features. The combined radiomics model showed the highest performance, with the LR model achieving an AUC of 0.824 ± 0.073 (95% CI: 0.809–0.838) (as in Fig 6). Statistical analysis demonstrated that the tumor model outperformed the mesorectal fat model (p-value = 1.96 × 10^−11^), while the combined model remained significantly superior to both individual models (p-value = 2.19 × 10^−4^ compared with the tumor model and p-value = 5.73 × 10^−15^ compared with the mesorectal fat model), as shown in Fig 7.

## Discussion

The findings of this study highlight the potential of MRI-based radiomics models which can improve the prediction of residual EMVI and PLN metastasis in locally advanced rectal cancer (LARC) after neoadjuvant chemoradiotherapy. Notably, the combined radiomics model incorporating features from both tumor and mesorectal fat achieved the best performance, highlighting the importance of evaluating not only the primary tumor but also the surrounding mesorectal compartment.

In the post-treatment setting, accurate identification of residual high-risk features is particularly important, as treatment-related fibrosis and tumor regression can obscure viable disease on conventional MRI. Our findings suggest that radiomics may provide additional value beyond visual assessment by capturing subtle quantitative features that reflect residual tumor biology. This is especially relevant in the current era of organ-preserving strategies, where reliable detection of residual disease is critical for appropriate patient selection.

From EMVI model performance, it was observed that the MRI-based radiomics model incorporating mesorectal fat features improves EMVI prediction in LARC patients. According to a literature review by Kim et al., [19] EMVI detection from MRI images interpreted by radiologists achieved an average sensitivity of 0.61 and specificity of 0.87. In contrast, our combined radiomics models demonstrated a sensitivity of 0.8 and specificity of 0.67, indicating that the combined radiomics model could serve as a helpful screening tool for EMVI prediction.

Interestingly, the radiomic feature wavelet-LLL_glszm_SmallAreaLowGrayLevelEmphasis was the only feature selected by both the mesorectal fat and tumor radiomics models in the Logistic Regression model. This suggests that this wavelet-based texture feature may play an important role in EMVI prediction. In the mesorectal fat radiomics model, two features; wavelet-HHH_firstorder_Mean and log-sigma-4–0-mm-3D_firstorder_90Percentile were consistently selected by all three models (LR, SVM, and RF). In the tumor radiomics model, the feature wavelet-HHL_gldm_DependenceNonUniformityNormalized was selected by all models. These results show that mesorectal fat and tumor regions provide different but complementary information for EMVI prediction and using features from both regions together improves predictive performance compared to analyzing only one region.

Compared to the study by Lin et al. [34], their best radiomics model for predicting EMVI from the tumor region was the SVM model, achieving an AUC of 0.981 on the training set and 0.861 on the validation set. In our study, the best model for the tumor region was the LR model, with an AUC of 0.846 on the training set and 0.757 ± 0.079 on the validation set. For the mesorectal fat model, our LR model achieved an AUC of 0.851 ± 0.017 on the training set and 0.776 ± 0.073 on the validation set. The combined model in our study showed improved performance, with an AUC of 0.930 ± 0.012 on the training set and 0.797 ± 0.073 on the validation set. These results emphasize that radiomic features from the mesorectal fat region can effectively predict EMVI, regardless of the type of model used, highlighting its value as an important biomarker.

For PLN prediction, MRI is neither sensitive nor specific in evaluation of PLN status in LARC patient, particularly in the setting of post NCRT. However, radiomics has shown potential for PLN prediction in colorectal cancer. From the literature review by Abbaspour E. et al [35], image-based radiomics models achieved an average AUC of 0.81 with a sensitivity of 0.77 and specificity of 0.734. In our study, the combined radiomics model demonstrated improved performance with an AUC of 0.824, sensitivity of 0.857, and specificity of 0.667. These results highlight that integrating mesorectal fat and tumor features improves both accuracy and sensitivity for PLN prediction compared to tumor-only models, suggesting its potential as a screening tool.

The radiomic feature wavelet-LHH_glrlm_LongRunLowGrayLevelEmphasis was the only feature consistently selected across all three models in the mesorectal fat radiomics model for PLN prediction. For the tumor radiomics model, two features—wavelet-LLH_glrlm_RunEntropy and wavelet-LLH_glcm_JointEnergy—were selected. These findings indicate that distinct radiomic features from mesorectal fat and tumor regions contribute valuable information for PLN prediction. Combining radiomic features from both regions provides a synergistic advantage, improving predictive accuracy for PLN.

As we developed three radiomics models (LR, SVM, and RF), we were able to identify radiomic features that may be key predictors of EMVI and PLN statuses. These findings highlight specific features that could serve as important biomarkers for predicting disease progression in LARC patients.

According to Jayprakasam et al. [29], their radiomics model for predicting pathological nodal status using features derived from mesorectal fat alone achieved an AUC of 0.74, whereas our study uniquely combines tumor and mesorectal fat radiomics features demonstrated an AUC of 0.758 for the mesorectal fat model and an improved AUC of 0.824 for the combined model. However, a notable difference lies in the number of selected radiomic features. Jayprakasam et al. selected only 8 features for their model, whereas our study utilized 16 features from mesorectal fat and 33 features in the combined model. This highlights the importance of optimizing the number of selected features to improve model performance while retaining essential information. Furthermore, their study excluded EMVI as a predictive region, whereas our findings demonstrate that mesorectal fat alone can predict both PLN and EMVI status. These results underlining the significant role of mesorectal fat as a biomarker for predicting multiple pathological statuses in LARC patients, including PLN and EMVI.

Our study aims to develop and evaluate a combined radiomics model that incorporatd features from both mesorectal fat and tumor regions. While most radiomics research in LARC has primarily focused on the tumor itself [22–25], mesorectal fat, one of the areas examined during mesorectal excision, has been found to be associated with poor outcomes and important prognostic value in LARC patients. [26–28]. Including radiomic features from both the tumor and surrounding mesorectal fat regions provides a better predictive radiomics model for predicting EMVI and PLN statuses, as shown in this study. This approach captures more information from the tumor microenvironment, which plays an important role in cancer prognosis. [36–39]. We also developed three different models (LR, SVM, and RF) to identify radiomic features that strongly associate with PLN and EMVI statuses. These findings highlight specific features that could be an important biomarker and emphasize the importance of mesorectal fat radiomics predicting pathological statuses in LARC patients. This study shows the importance of combining MRI-based radiomics from mesorectal fat and tumor regions to enhance the prediction of EMVI and PLN statuses in LARC patients. Our combined radiomics models demonstrated better predictive performance, with the potential to aid in therapeutic decision-making. These findings support the role of mesorectal fat as the crucial tumor microenvironment, providing essential prognostic information [40].

Moreover, our study reinforces that mesorectal fat radiomics independently provides valuable prognostic insights, regardless of the prediction model used. This aligns with emerging evidence that peritumoral tissues contain valuable biomarkers reflecting the interaction between tumor progression and the surrounding microenvironment [41–44].

Importantly, our study was designed to address a clinically relevant question in the post-nCRT setting, focusing on patient-level prediction of residual EMVI and nodal metastasis rather than lesion-level detection. This approach reflects real-world clinical decision-making, where treatment strategies—particularly the consideration of organ-preserving approaches such as watch-and-wait—are based on overall assessment of residual disease within the mesorectum. In this context, accurate identification of patients without residual high-risk features may support less aggressive management, whereas detection of residual EMVI or nodal disease may prompt surgical intervention or closer surveillance.

This study, however, had several limitations. Firstly, the focus was placed solely on perirectal lymph nodes, which are the closest nodes to the primary tumor and the most common initial site for metastasis in LARC. To further enhance PLN prediction, future research should explore radiomic features in additional lymph node regions. Secondly, the retrospective and single-cohort design of this study may restrict the generalizability of our findings. Although MRI images were acquired using different scanners which could introduce variability in image quality, we minimized this effect by resampling all images to the smallest available voxel size (0.5×0.5×0.5 mm³). As a result, the influence of scanner variation is expected to be minimal. Moreover, the limited number of LARC patients with positive EMVI and PLN statuses in our dataset hindered the ability to perform external validation. Moving forward, multi-institutional studies with larger, more diverse cohorts and external test sets are essential to validate the robustness of our models and confirm their clinical applicability. Additionally, exploring radiomics features from other perirectal regions may further enhance predictive performance.

## Conclusion

In conclusion, this study demonstrates that combined radiomic features derived from mesorectal fat and tumor regions significantly enhance the prediction of EMVI and PLN statuses in LARC patients who completed neoadjuvant chemoradiotherapy. The combined radiomics model, particularly using Logistic Regression, showed superior predictive performance compared to individual models. The key radiomic features from restaging MRI performed prior to surgery identified in this study provide important insights into the distinct contributions of these regions for prediction. While further validation is required, our findings suggest that combined radiomics models could serve as promising non-invasive tools for improving prognostic accuracy and supporting personalized treatment strategies in clinical practice.

## Supporting information

S1 File Supplementary1_Radiomics_Selected_Features_LR_EMVI. (XLSX)

S2 File Supplementary2_Radiomics_Selected_Features_LR_PLN. (XLSX)

S3 File Supplementary3_Radiomics_Selected_Features_RF_PLN. (XLSX)

S4 File Supplementary4_Radiomics_Selected_Features_SVM_EMVI. (XLSX)

S5 File Supplementary5_Radiomics_Selected_Features_SVM_PLN. (XLSX)

S6 File Supplementary6_Radiomics_Selected_Features_RF_EMVI. (XLSX)

S7 File Supplementary7_delong_data_EMVI_LR. (XLSX)

S8 File Supplementary8_delong_data_PLN_LR. (XLSX)

S9 File Supplementary9_delong_data_EMVI_RF. (XLSX)

S10 File Supplementary10_delong_data_PLN_RF. (XLSX)

S11 File Supplementary11_delong_data_EMVI_SVM. (XLSX)

S12 File Supplementary12_delong_data_PLN_SVM. (XLSX)

S13 File Supplementary13_AUC_AllModelEMVI. (XLSX)

S14 File Supplementary14_AUC_AllModelPLN. (XLSX)

S15 Fig Overview of the analytical workflow. (PPTX)

S16 Fig CONSORT. (TIF)
